# Supplementary material for: Gaining more from doing less? The effects of a one-week deload period during supervised resistance training on muscular adaptations
Source: PeerJ. 2024 Jan 22;12:e16777. doi: 10.7717/peerj.16777 (PMC10809978; doi:10.7717/peerj.16777)
Supplement: Supplemental Information 1 [file peerj-12-16777-s001.docx]

Overview of the training protocol; participants performed the routine twice per week.

***Lower Body Routine**

| Exercise | Sets | Repetitions | Rest Interval |
| --- | --- | --- | --- |
| Smith machine squat | 5 | 8-12 | 2 min |
| Leg Extension | 5 | 8-12 | 2 min |
| Straight Knee Calf Raise | 5 | 8-12 | 2 min |
| Bent Knee Calf Raise | 5 | 8-12 | 2 min |

**^#^Upper Body Routine**

| Exercise | Sets | Repetitions | Rest Interval |
| --- | --- | --- | --- |
| Shoulder Press | 5 | 8-12 | 2 min |
| Lat Pulldown | 5 | 8-12 | 2 min |
| Chest Press | 5 | 8-12 | 2 min |
| Biceps Curl | 5 | 8-12 | 2 min |
| Triceps Pushdown | 5 | 8-12 | 2 min |

*Supervised training; ^#^Unsupervised training
