# Supplementary material for: Gaining more from doing less? The effects of a one-week deload period during supervised resistance training on muscular adaptations
Source: PeerJ. 2024 Jan 22;12:e16777. doi: 10.7717/peerj.16777 (PMC10809978; doi:10.7717/peerj.16777)
Supplement: Supplemental Information 2 [file peerj-12-16777-s002.pdf]

Answers to readiness-to-train (RTQ) question

The questionnaire consists of seven questions with responses made from bipolar scales ranging from 1 to 4, 1 to 5 and 1 to 10.

1 can be described as not at all/extremely low and 4, 5, 10 (depending on lower/upper end of the scale) can be described as extreme amount/extremely high.

Q1: Do you feel physically strong today? (1-5)	1	2	3	4	5
Q2: Do you feel mentally strong today? (1-5)	1	2	3	4	5
Q3: How would you describe your health today? (1-4)	1	2	3	4	
Q4: How would you describe your appetite over the past 24h? (1-5)	1	2	3	4	5
Q5: How would you describe you sleep quality over the past 24h? (1-4)	1	2	3	4	

Q6: Do you have any muscle soreness today? (1-10)	1	2	3	4	5	6	7	8	9	10
Q7: Rate your motivation to train today (1-10)	1	2	3	4	5	6	7	8	9	10
