# Supplementary material for: Gaining more from doing less? The effects of a one-week deload period during supervised resistance training on muscular adaptations
Source: PeerJ. 2024 Jan 22;12:e16777. doi: 10.7717/peerj.16777 (PMC10809978; doi:10.7717/peerj.16777)
Supplement: Supplemental Information 3 [file peerj-12-16777-s003.pdf]

Supplementary File

The following supplementary file provides additional analyses and presents the WAMBS (When to worry, and how to Avoid the Misuse of Bayesian Statistics) checklist as a diagnostic tool that was used to assess prior distributions, the estimation process, and the influence of priors for analysis of body composition and performance outcomes [1]. The following provides details of the WAMBS checklist and how it was used.

THE WAMBS-CHECKLIST

When to worry, and how to Avoid the Misuse of Bayesian Statistics

DEPAOLI & VAN DE SCHOOT (2016)

**TO BE CHECKED BEFORE ESTIMATING**	
**Point 1:** Do you understand the priors?	Analyses were conducted using ANCOVAs with the pre-intervention value centered. The intercept therefore represents the post-intervention value. Informative priors from S&C research were used to identify the post value based on typical change from pre-intervention [2]. Values were obtained on the original scale by multiplying by the pooled pre-intervention standard deviation. For the group effect, informative priors were centered on zero, with the standard deviation based on variation observed in comparative research in S&C [3]. Actual prior distributions are provided for each model.
**TO BE CHECKED AFTER ESTIMATION**	
**Point 2:** Does the trace-plot exhibit convergence?	Trace-plots were examined and all Rhat values were reported.
**Point 3:** Does convergence remain after doubling the number of iterations?	Trace-plots were examined and bias for group parameter presented as a percentage 100*(original – doubling)/original reported.
**Point 4:** Does the histogram have enough information?	Plot of histogram for all parameters presented.
**Point 5:** Do chains exhibit autocorrelation?	Plot of autocorrelation for all parameters presented.
**Point 6:** Do posterior distributions make sense?	In all cases yes
**UNDERSTANDING INFLUENCE OF PRIORS**	
**Point 7:** Do different variance priors influence the results?	Sigma was modeled using weakly-informative Half-t distributions with 3df. As a check, informative gamma priors with shape k based on outcome and scale θ set to 1. Bias in group parameter was presented.
**Point 8:** Is there a notable effect of the prior when compared with non-informative priors?	As a check, models were conducted with all default weakly-informative priors and bias in group parameter presented.
**Point 9:** Are the results stable from a sensitivity analysis?	Checked in each case, use of informative priors tended to reduce point estimate and tails of group parameter.
**AFTER INTERPRETATION OF RESULTS**	
**Point 10**: Is the Bayesian way of interpreting and reporting model results used? *(a) Also report on: missing data, model fit and comparison, non-response, generalizability, ability to replicate, etc.*	Reporting of results combining in-text and supplementary file was done in a comprehensive and systematic manner, focussing on the group difference parameter and the magnitude relative to thresholds specific to S&C. Posterior probabilities were also used to summarize likely differences between groups.

References

1. Depaoli S, van de Schoot R. Improving transparency and replication in bayesian statistics: The WAMBS-checklist. Psychol Methods. 2017 June 01;22(2):240-61.
2. Swinton PA, Burgess K, Hall A, Greig L, Psyllas J, Aspe R, et al. Interpreting magnitude of change in strength and conditioning: Effect size selection, threshold values and bayesian updating. J Sports Sci. 2022 September 01;40(18):2047-54.
3. Swinton PA, Murphy A. Comparative effect size distributions in strength and conditioning and implications for future research: A meta-analysis. SportRxiv. 2022:DOI: 10.51224/SRXIV.202.

Body composition

Muscle thickness

1) <u>Rectus femoris</u>

Measurements were obtained at 30%, 50% and 70% between the lateral condyle of the femur and greater trochanter.

Individual data points

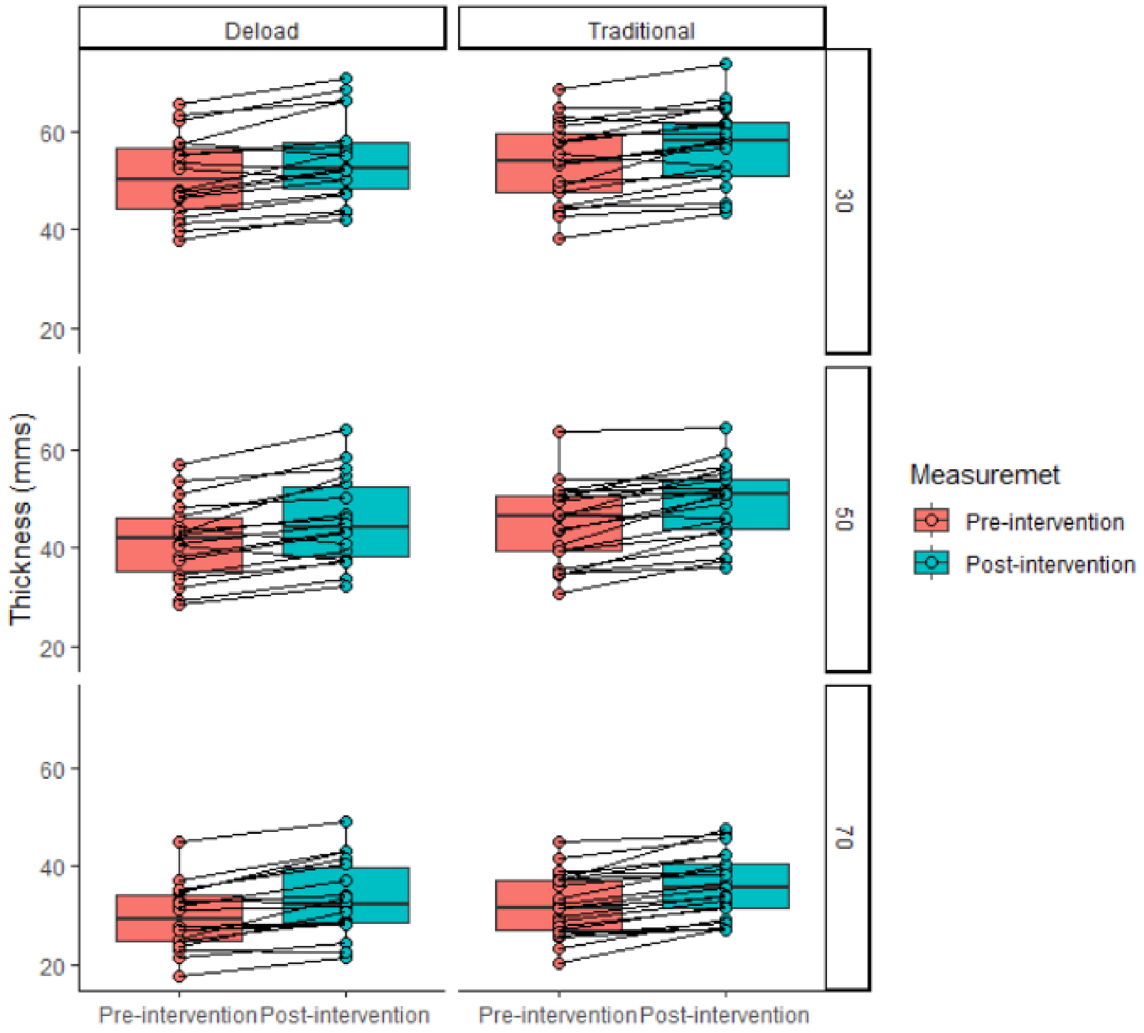

WAMBS: Univariate rectus femoris thickness (30/50/70)

Understanding priors

Outcome	Rectus femoris 30%	Rectus femoris 50%	Rectus femoris 70%
Intercept	Normal(56.0,3.6^2)	Normal(46.7,3.6^2)	Normal(33.8,3.6^2)
Centered Pre Value	Normal(0.85,2.0^2)	Normal(0.85,2.0^2)	Normal(0.85,2.9^2)
Group	Normal(0,3.6^2)	Normal(0,3.6^2)	Normal(0,3.6^2)
Prior predictive check	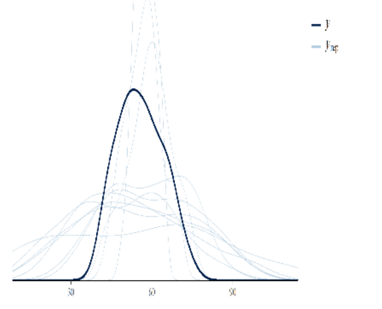	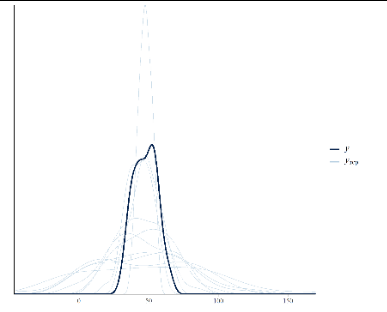	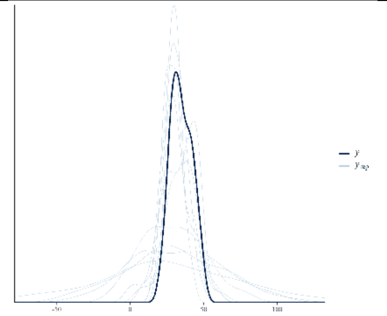

Estimation

Outcome	Rectus femoris 30%	Rectus femoris 50%	Rectus femoris 70%
Rhat values	All equal 1.0	All equal 1.0	All equal 1.0
Bias doubling iterations (Group)	1.7%	-1.6%	5.5%
Posterior predictive check	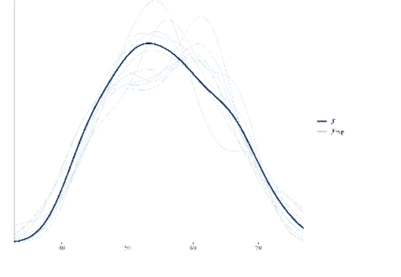	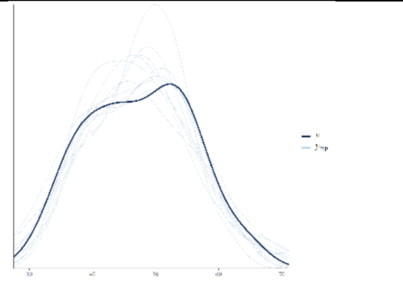	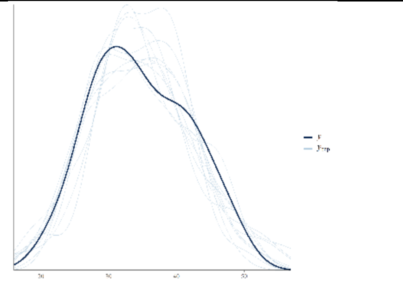
Histogram and correlation	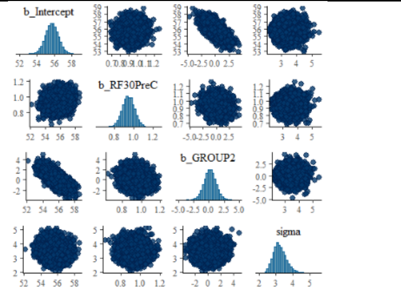	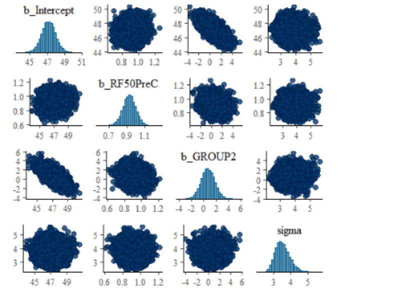	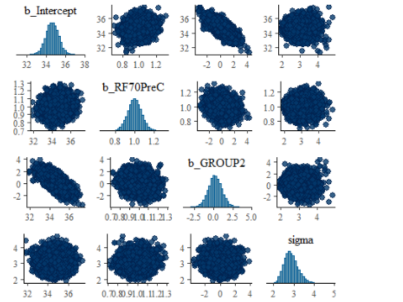
Autocorrelation	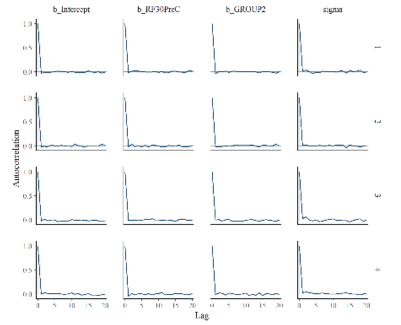	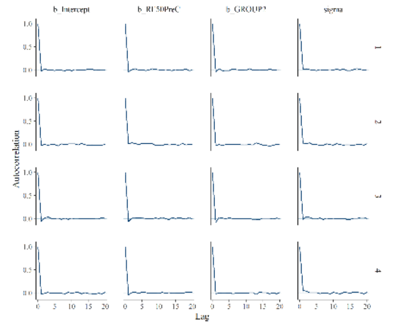	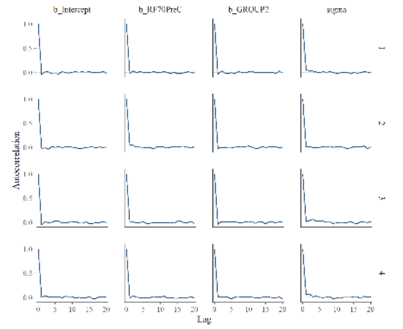

Influence of priors

Outcome	Rectus femoris 30%	Rectus femoris 50%	Rectus femoris 70%
Bias different specification variance (Group)	Gamma(3,1)<br>8.1%	Gamma(3,1)<br>-3.5%	Gamma(3,1)<br>-1.7%
Bias after non-informative priors (Group)	-4.1%	-11.1%	-24.7%

2) <u>Vastus lateralis</u>

Measurements were obtained at 30%, 50% and 70% between the lateral condyle of the femur and greater trochanter.

Individual data points

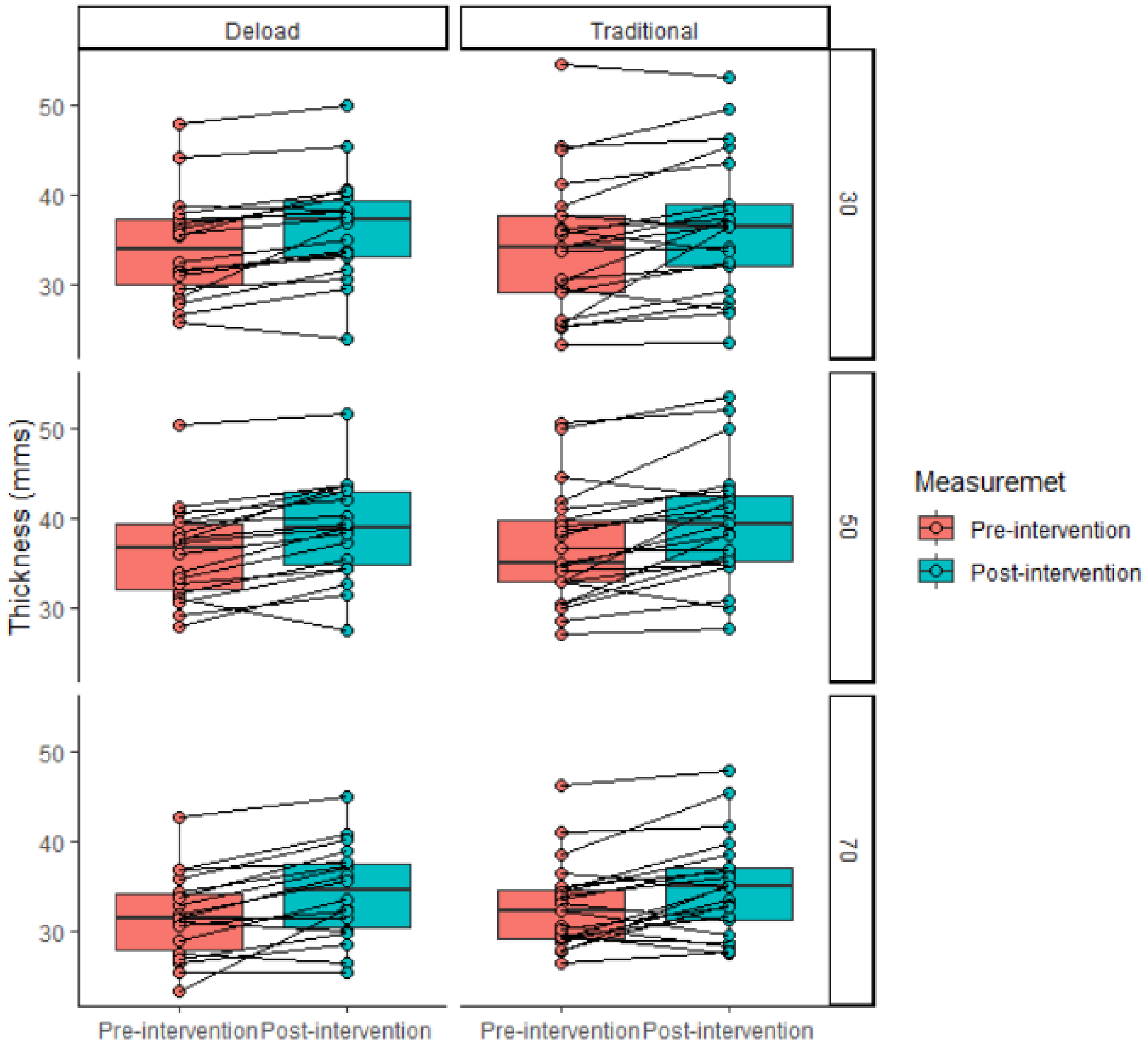

WAMBS: Vastus lateralis thickness (30/50/70)

Understanding priors

Outcome	Vastus lateralis 30%	Vastus lateralis 50%	Vastus lateralis 70%
Intercept	Normal(37.3.0,3.1^2)	Normal(38.9,2.6^2)	Normal(34.3,2.1^2)
Centered Pre Value	Normal(0.85,2.0^2)	Normal(0.85,2.0^2)	Normal(0.85,2.9^2)
Group	Normal(0,2.8^2)	Normal(0,2.4^2)	Normal(0,1.9^2)
Prior predictive check	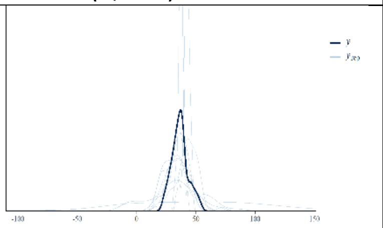	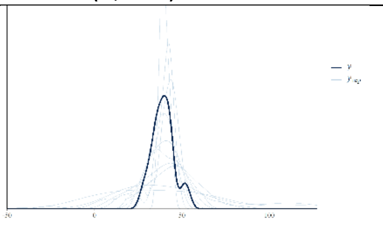	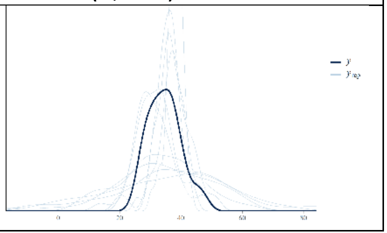

Estimation

Outcome	Vastus lateralis 30%	Vastus lateralis 50%	Vastus lateralis 70%
Rhat values	All equal 1.0	All equal 1.0	All equal 1.0
Bias doubling iterations (Group)	-11.6%	-1.6%	-1.0%
Posterior predictive check	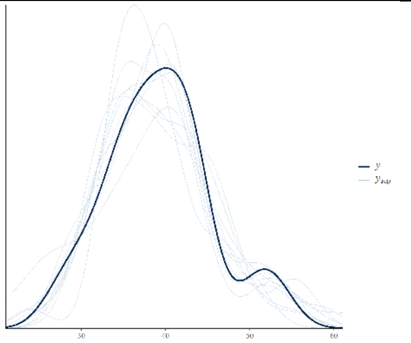	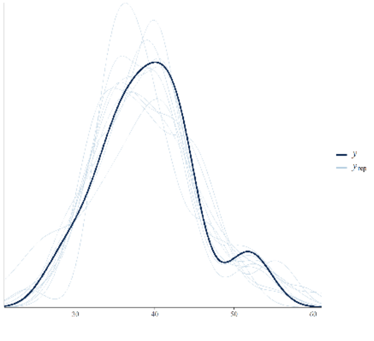	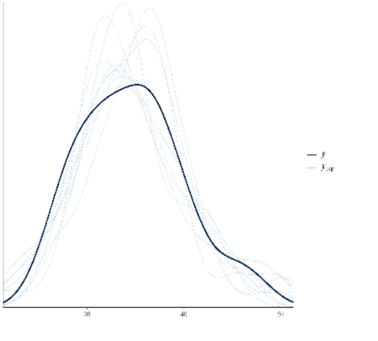
Histogram and correlation	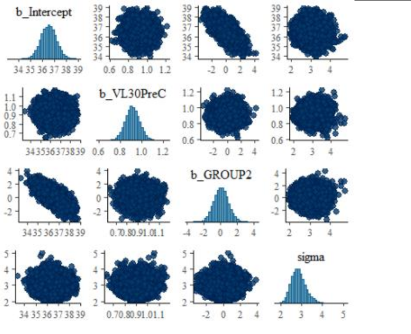	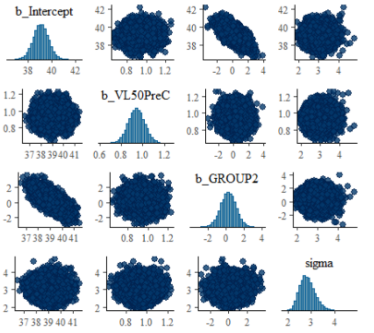	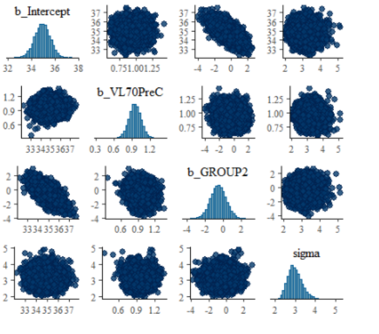
Autocorrelation	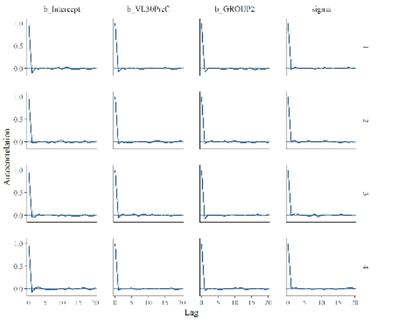	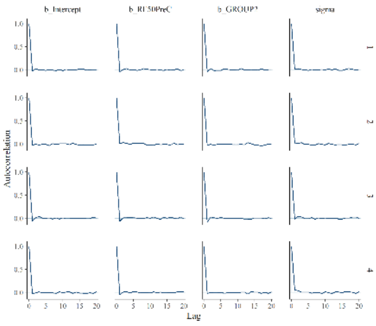	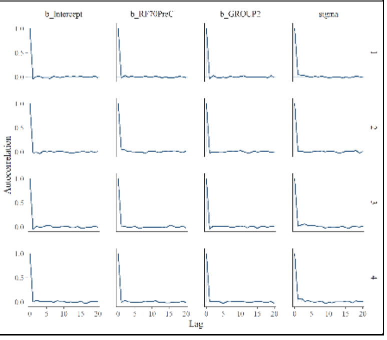

Influence of priors

Outcome	Vastus lateralis 30%	Vastus lateralis 50%	Vastus lateralis 70%
Bias different specification variance (Group)	Gamma(3,1)<br>-4.7%	Gamma(3,1)<br>2.3%	Gamma(3,1)<br>-1.7%

Bias after non-informative priors (Group)	-9.8%	-16.8%	-3.7%

3) <u>Calf thickness</u>

Outcomes included thickness of the lateral gastrocnemius, medial gastrocnemius, and soleus. Measurements were taken on the posterior surface of both legs at 25% of the lower leg length (the distance from the articular cleft between the femur and tibia condyles to the lateral malleolus).

Individual data points

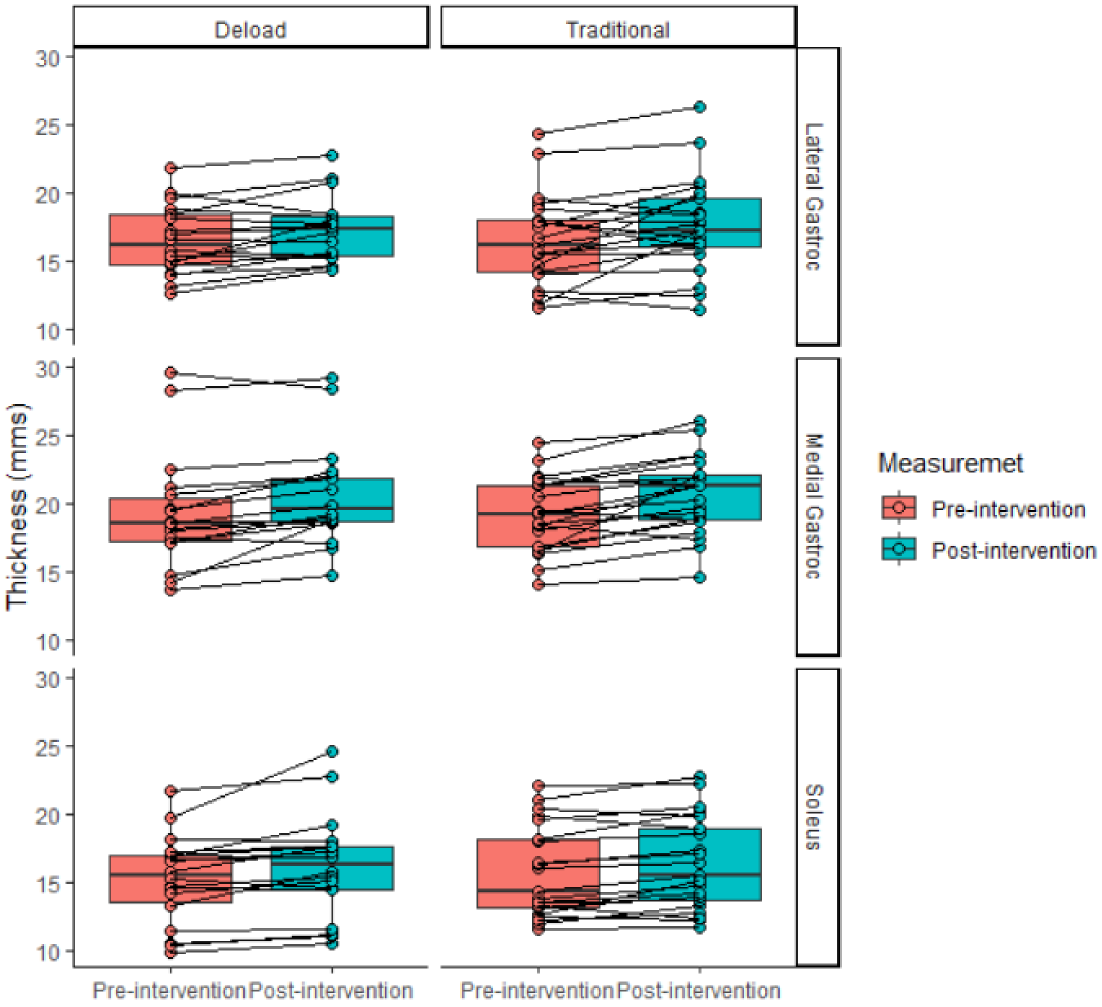

WAMBS: lateral gastrocnemius, medial gastrocnemius, soleus

Understanding priors

Outcome	Lateral gastrocnemius	Medial gastrocnemius	Soleus
Intercept	Normal$(20.7,1.5^2)$	Normal$(17.8,1.3^2)$	Normal$(16.9,1.4^2)$
Centered Pre Value	Normal$(0.85,2.0^2)$	Normal$(0.85,2.0^2)$	Normal$(0.85,2.9^2)$
Group	Normal$(0,1.3^2)$	Normal$(0,1.1^2)$	Normal$(0,1.3^2)$
Prior predictive check	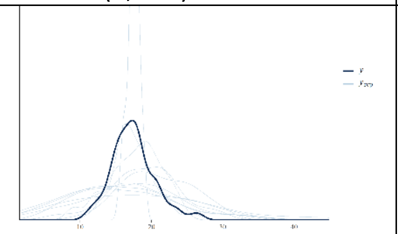	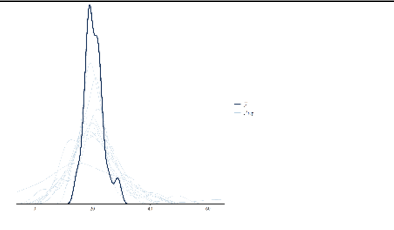	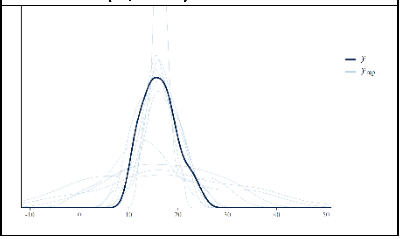

Estimation

Outcome	Lateral gastrocnemius	Medial gastrocnemius	Soleus
Rhat values	All equal 1.0	All equal 1.0	All equal 1.0
Bias doubling iterations (Group)	-4.2%	3.4%	2.0%
Posterior predictive check	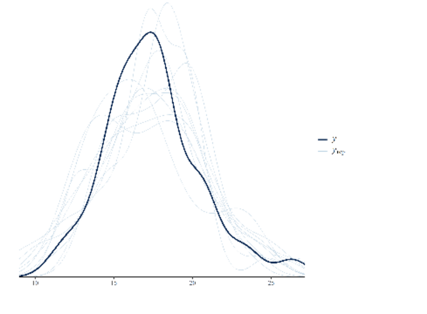	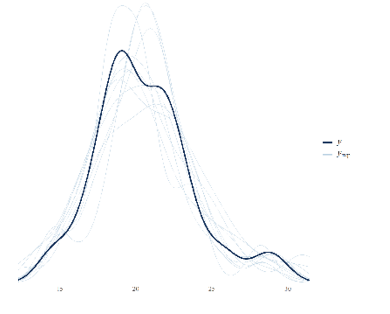	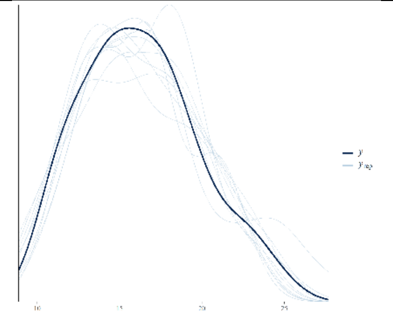
Histogram and correlation	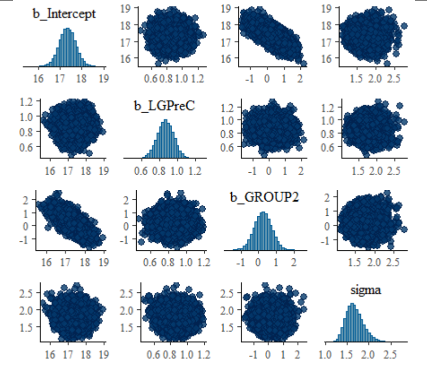	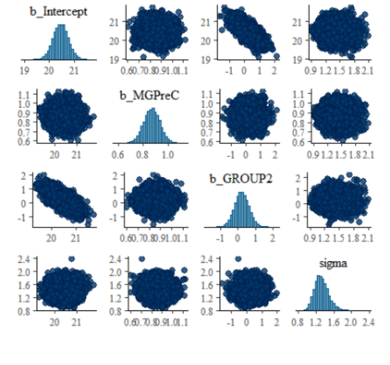	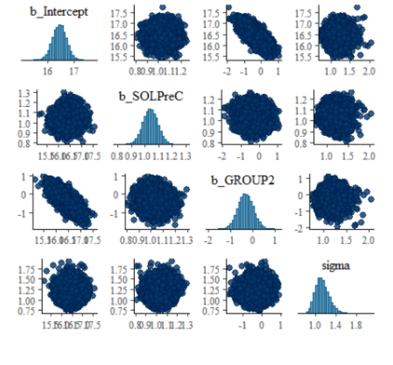
Autocorrelation	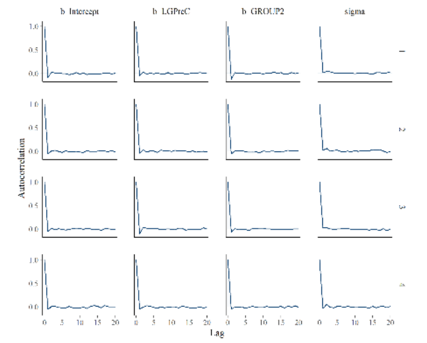	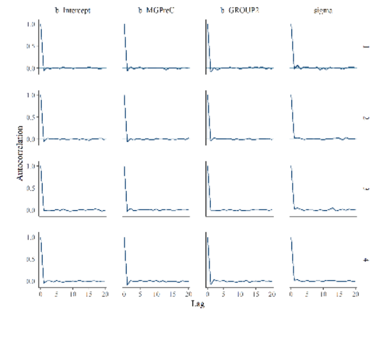	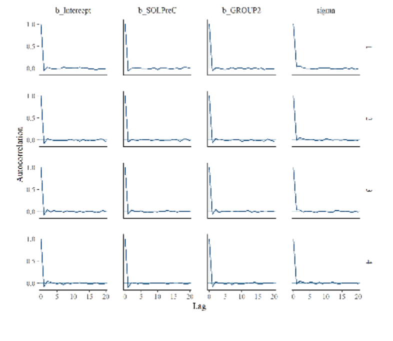

Influence of priors

Outcome	Lateral gastrocnemius	Medial gastrocnemius	Soleus
Bias different specification variance (Group)	Gamma(1,1)<br>-4.3%	Gamma(1,1)<br>4.5%	Gamma(1,1)<br>2.2%

Bias after non-informative priors (Group)	-22.5%	-7.2%	-3.8%

Body fat and lean mass

4) Lower body lean mass and body fat percentage

Lower body lean mass and body fat percentage were obtained by multifrequency bioelectrical impedance analysis.

Individual data points

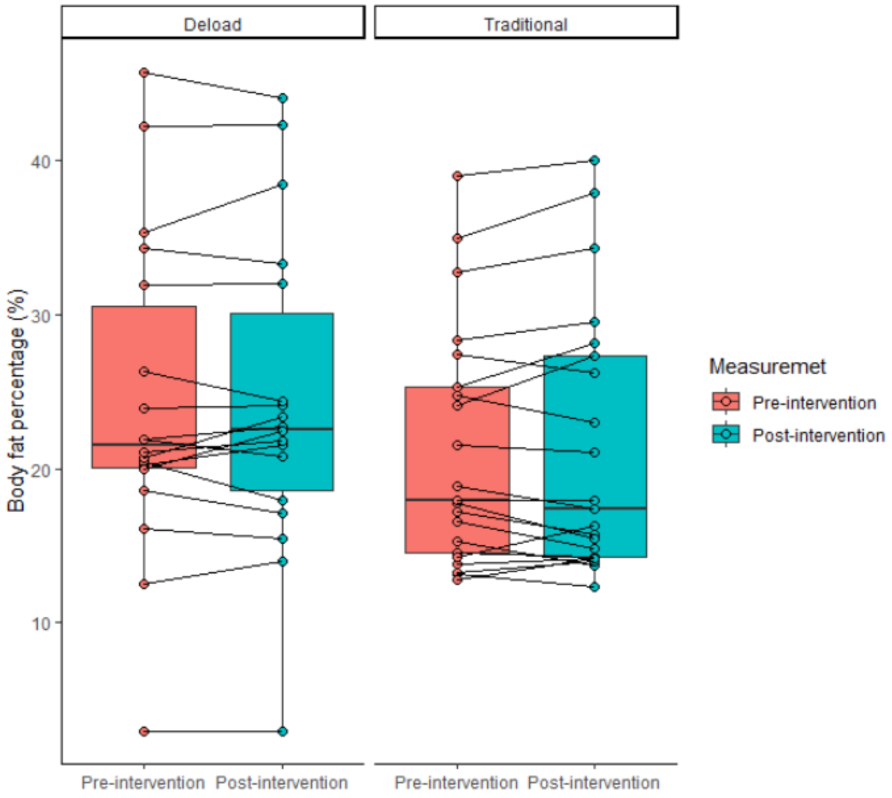

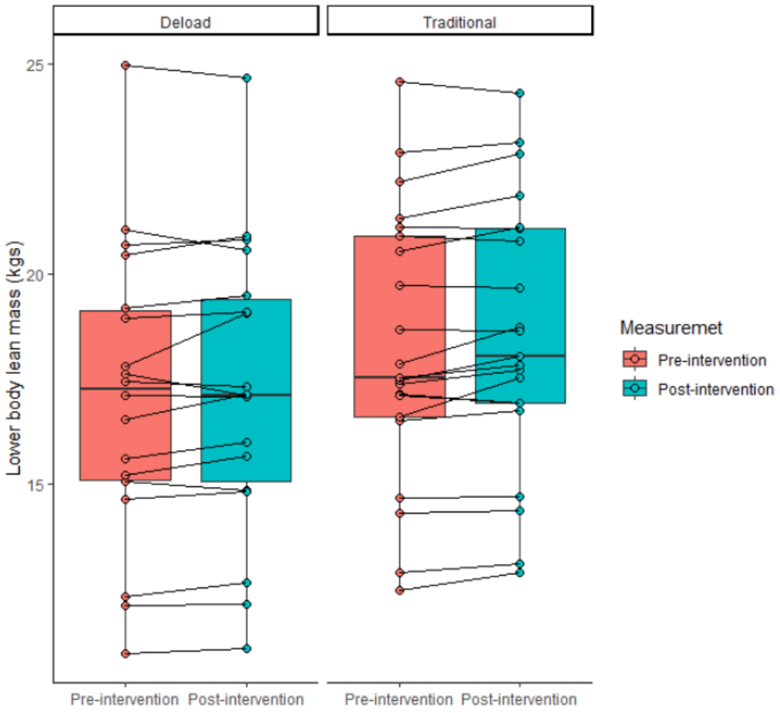

Deload
Traditional
25
20
15
Lower body lean mass (kgs)
Pre-intervention
Post-intervention
Pre-intervention
Post-intervention
Measuremet
Pre-intervention
Post-intervention

WAMBS: Body fat percentage and lower body lean mass

Understanding priors

Outcome	Body fat %	Lower body lean mass
Intercept	Normal(26.6,4.0^2)	Normal(42.7,3.3^2)
Centered Pre Value	Normal(0.85,2.0^2)	Normal(0.85,2.0^2)
Group	Normal(0,3.5^2)	Normal(0,2.9^2)
Prior predictive check	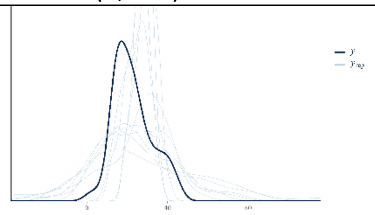	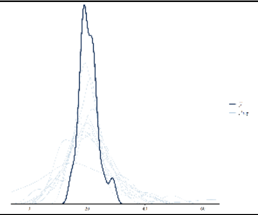

Estimation

Outcome	Body fat %	Lower body lean mass
Rhat values	All equal 1.0	All equal 1.0
Bias doubling iterations (Group)	-4.2%	-15.7%
Posterior predictive check	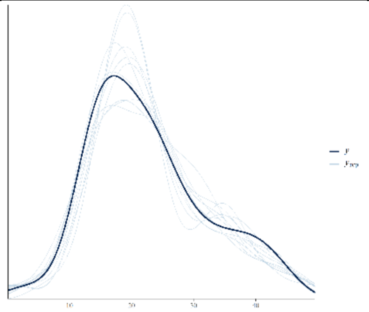	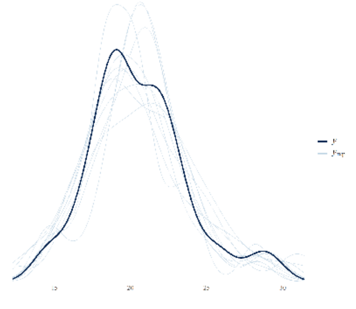
Histogram and correlation	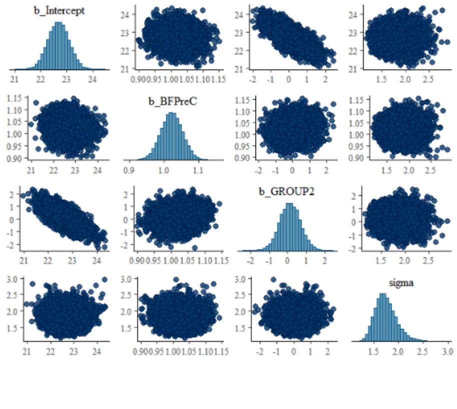	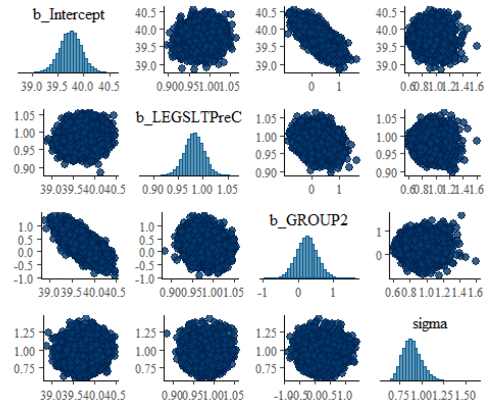
Autocorrelation	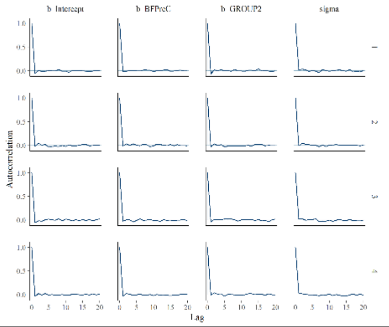	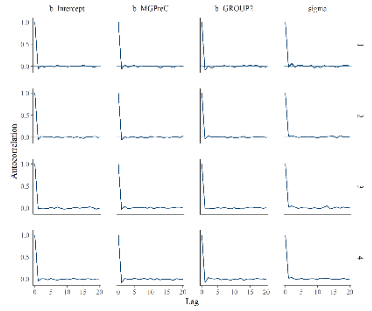

Influence of priors

Outcome	Body fat %	Lower body lean mass
Bias different specification variance (Group)	Gamma(1,1)<br>-6.3%	Gamma(1,1)<br>4.5%

Bias after non-informative priors (Group)	-19.9%	-7.2%

Body composition within group effect size summary

Bayesian standardized mean difference effect sizes were estimated accounting for uncertainty in mean change for each group and pre-intervention standard deviation.

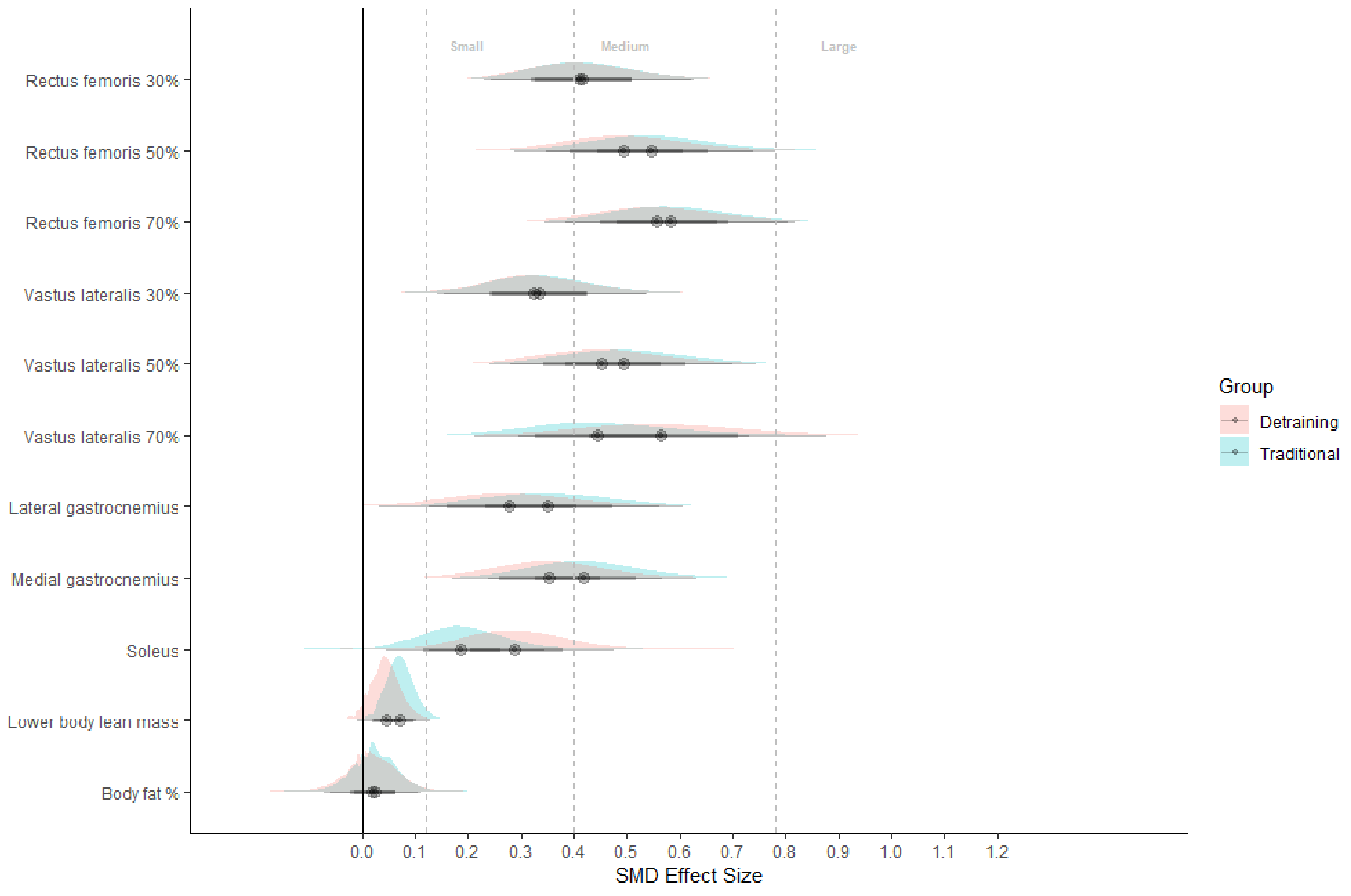

Small (0.12), medium (0.40) and large (0.78) thresholds derived for strength and conditioning interventions are presented with gray lines.

Performance

Strength

1) <u>Isometric and dynamic strength</u>

Isometric strength assessment was carried out using dynamometry testing with the participant seated and performing unilateral isometric actions of the knee extensors of the dominant limb. Dynamic lower body strength was assessed by 1RM testing in the back squat exercise performed on a Smith machine.

Individual data points

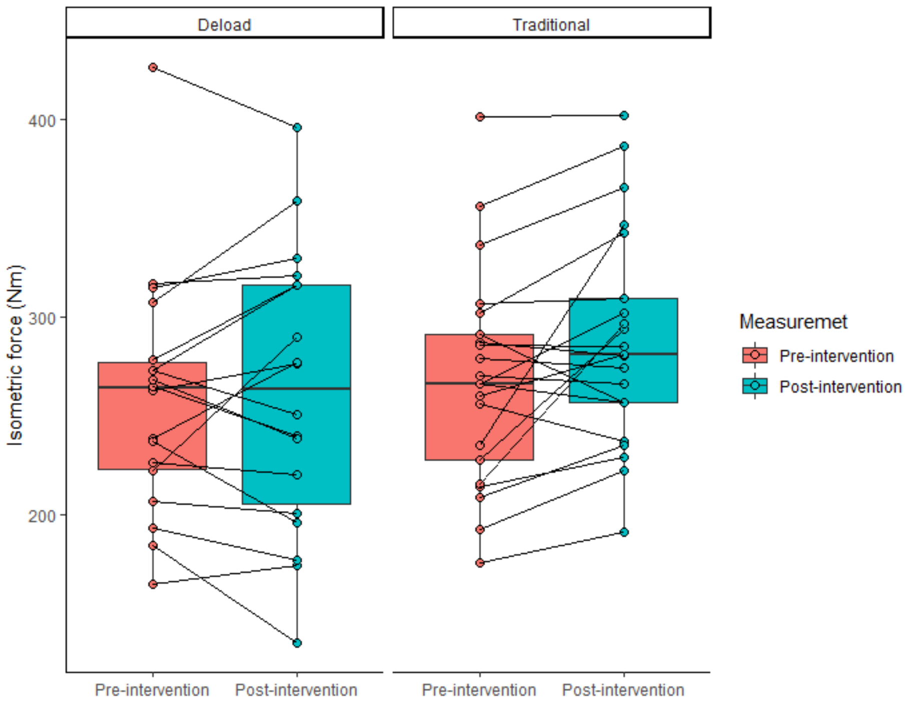

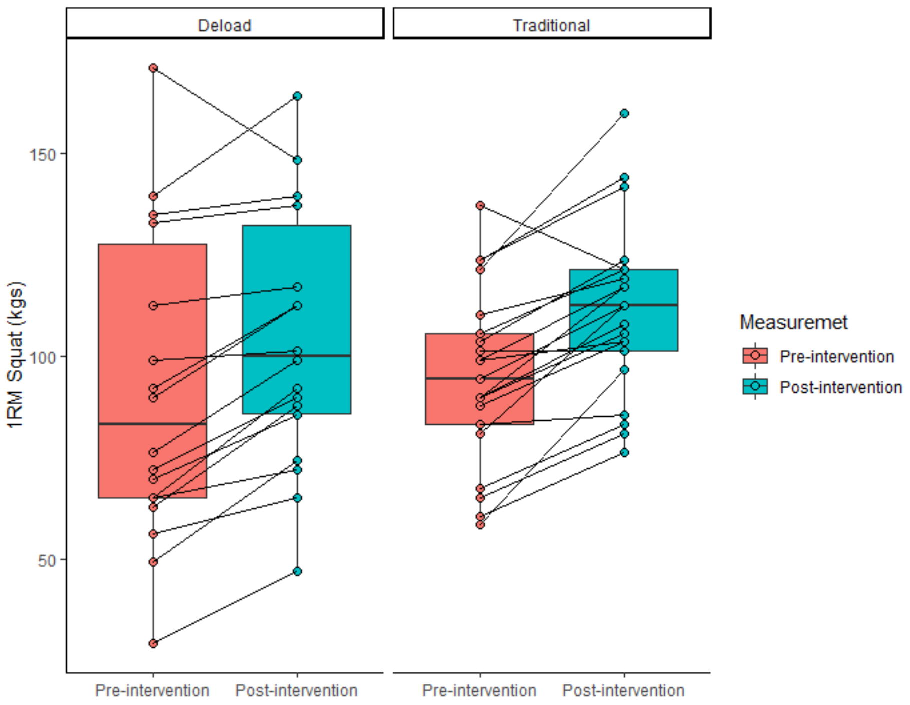

WAMBS: Isometric and dynamic strength

Understanding priors

Outcome	Isometric strength	Dynamic strength
Intercept	Normal(289.0,25.1^2)	Normal(237.0,29.3^2)
Centered Pre Value	Normal(0.85,2.0^2)	Normal(0.85,2.0^2)
Group	Normal(0,22.8^2)	Normal(0,26.6^2)
Prior predictive check	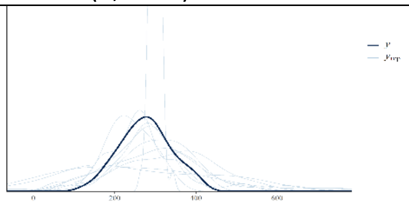	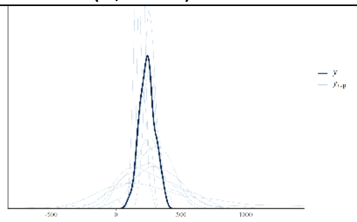

Estimation

Outcome	Isometric strength	Dynamic strength
Rhat values	All equal 1.0	All equal 1.0
Bias doubling iterations (Group)	-1.8%	-1.4%
Posterior predictive check	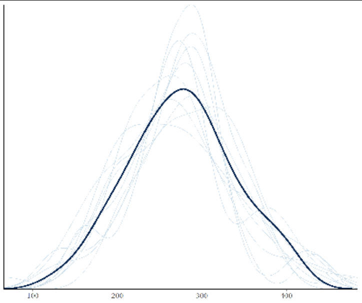	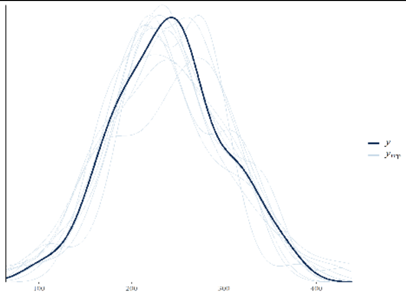
Histogram and correlation	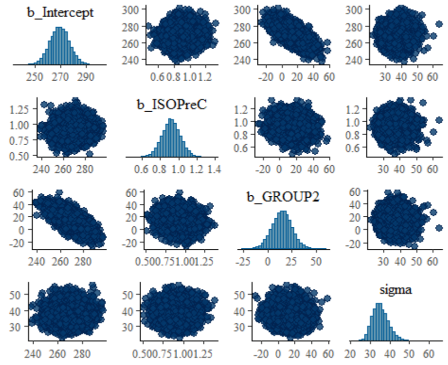	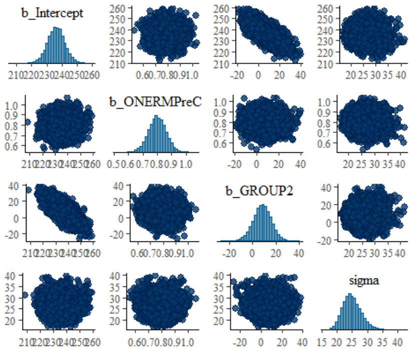
Autocorrelation	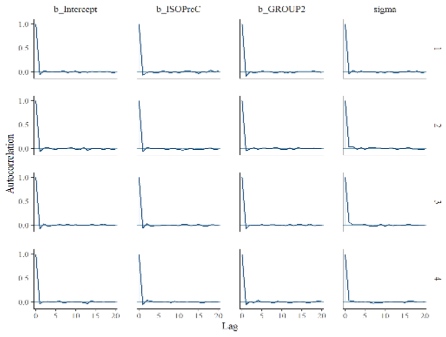	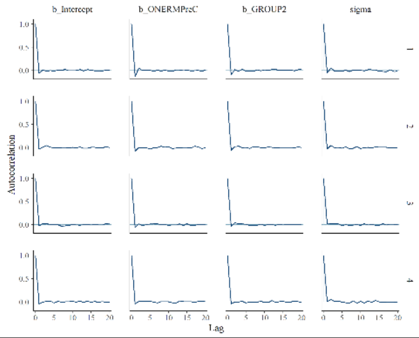

Influence of priors

Outcome	Isometric strength	Dynamic strength
Bias different specification variance (Group)	Gamma(25,1)<br>-2.7%	Gamma(25,1)<br>-2.0%
Bias after non-informative priors (Group)	-25.5%	-10.9%

Power and endurance

2) <u>Lower body muscle power and strength endurance</u>

Lower body muscle power was assessed via the vertical jump test performed via a countermovement with hands on hips attempting to jump as high as possible. Lower-body muscular strength-endurance was assessed by performing the leg extension exercise using 60% of the participant's initial body mass for as many repetitions as possible maintaining a constant cadence of 1-0-1-0 as monitored by a metronome.

Individual data points

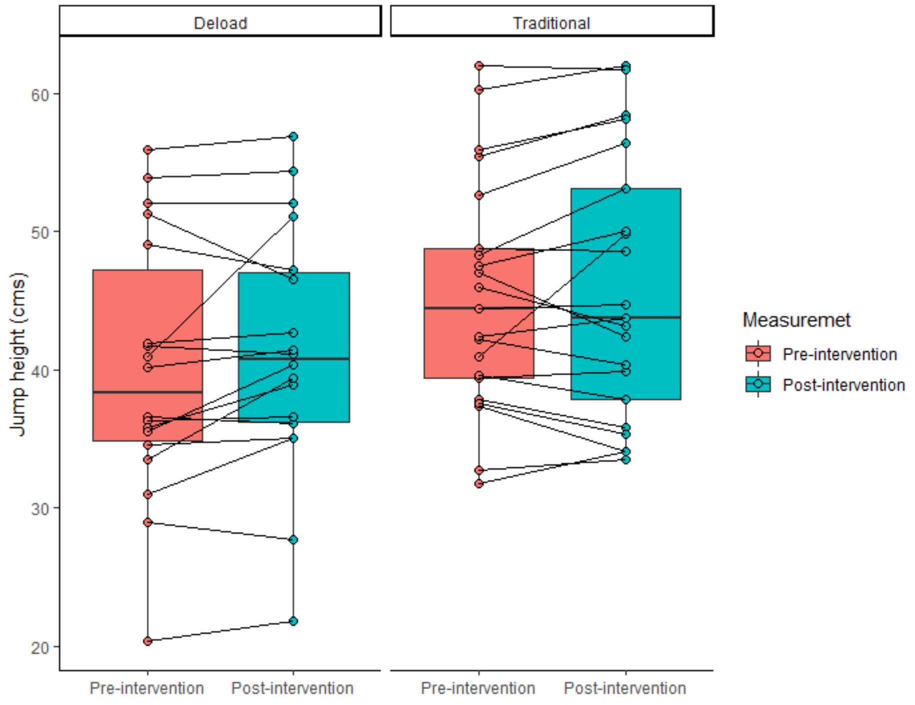

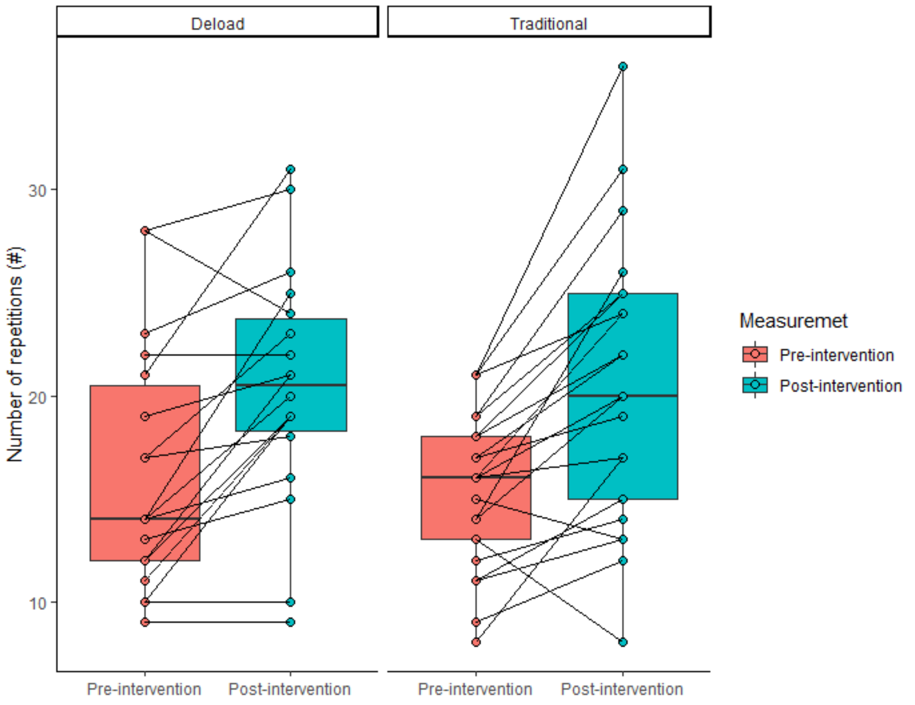

WAMBS: Muscle power and strength endurance

Understanding priors

Outcome	Muscle power	Strength endurance
Intercept	Normal(18.4,1.6^2)	Normal(18.0,2.2^2)
Centered Pre Value	Normal(0.85,2.0^2)	Normal(0.85,2.0^2)
Group	Normal(0,1.4^2)	Normal(0,2.0^2)
Prior predictive check	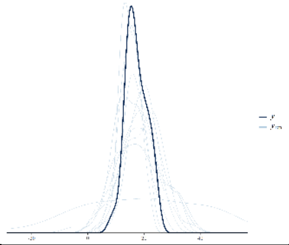	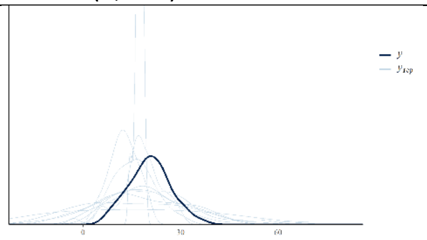

Estimation

Outcome	Muscle power	Strength endurance
Rhat values	All equal 1.0	All equal 1.0
Bias doubling iterations (Group)	1.8%	-0.1%
Posterior predictive check	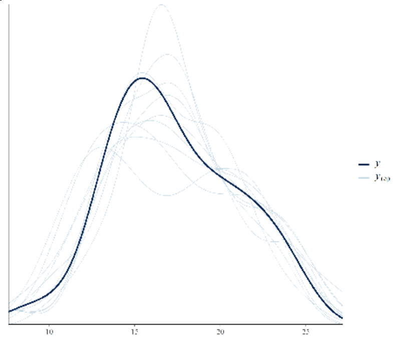	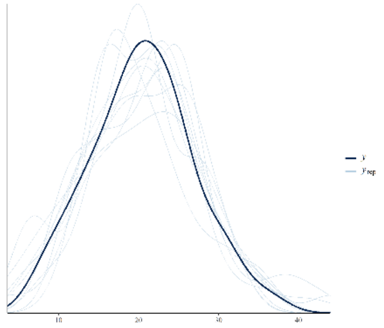
Histogram and correlation	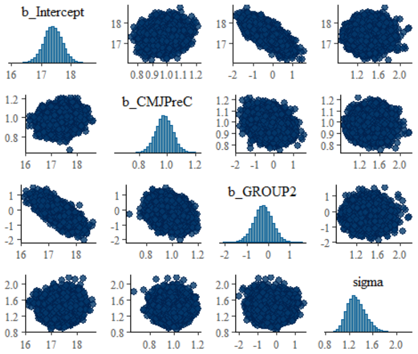	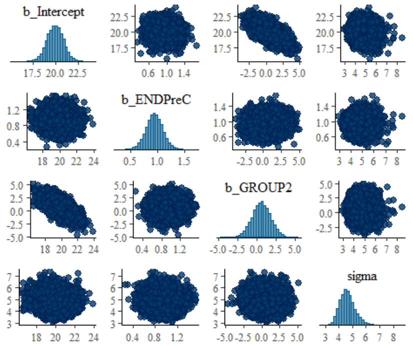
Autocorrelation	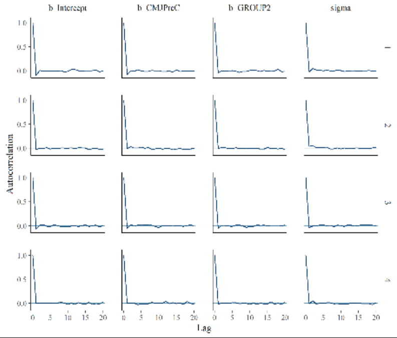	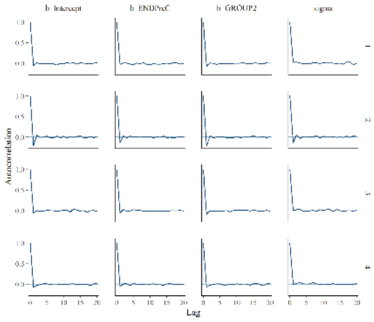

Influence of priors

Outcome	Muscle power	Strength endurance
Bias different specification variance (Group)	Gamma(1,1)<br>3.0%	Gamma(5,1)<br>-4.8%

Bias after non-informative priors (Group)	-3.0%	-3.5%

Performance within group effect size summary

Bayesian standardized mean difference effect sizes were estimated accounting for uncertainty in mean change for each group and pre-intervention standard deviation.

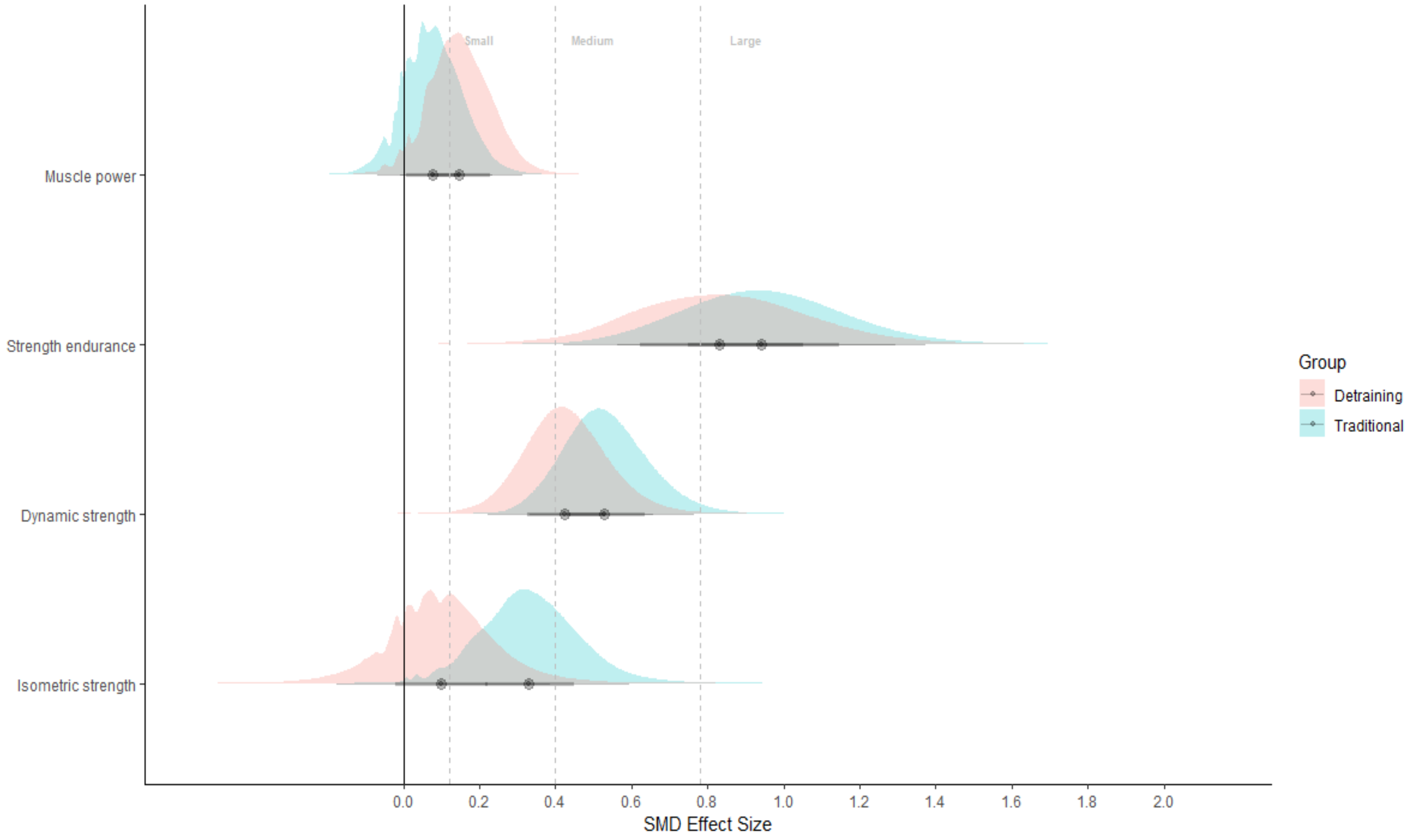

Small (0.12), medium (0.40) and large (0.78) thresholds derived for strength and conditioning interventions are presented with gray lines.

Nutritional intake

Dietary adherence was assessed by self-reported 5-day food records (including at least 1 weekend day) pre- and post-intervention using MyFitnessPal.com and comprised total energy consumption, as well as the amount of energy derived from proteins, fats, and carbohydrates. This section presents the individual data points and analyses to investigate potential changes across the intervention and between groups.

Individual data points

1) Daily energy intake

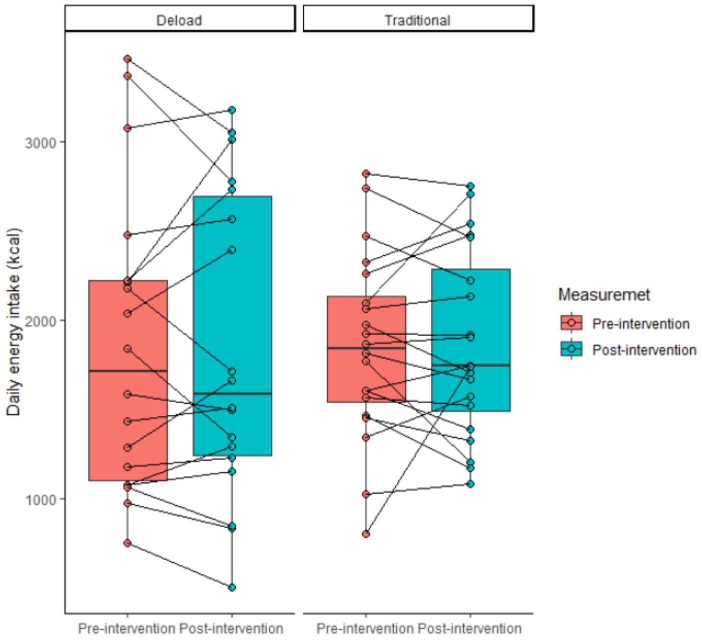

2) Carbohydrate intake

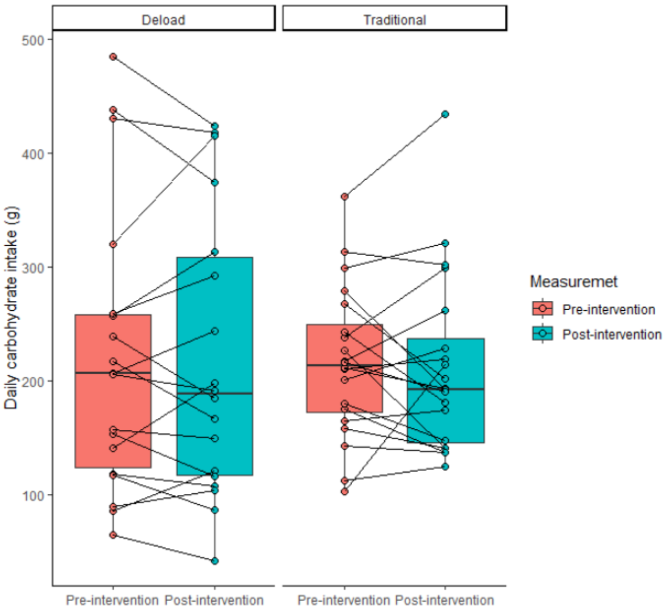

3) Fat intake

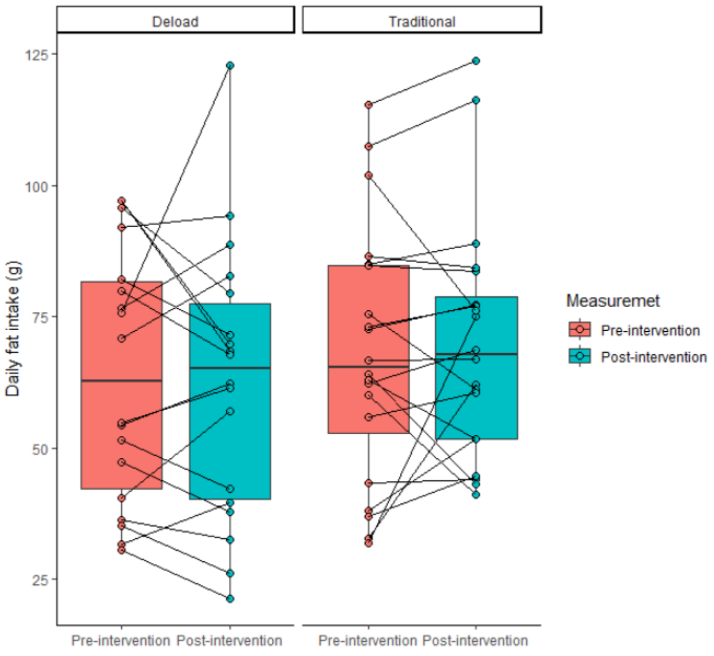

4) Protein intake

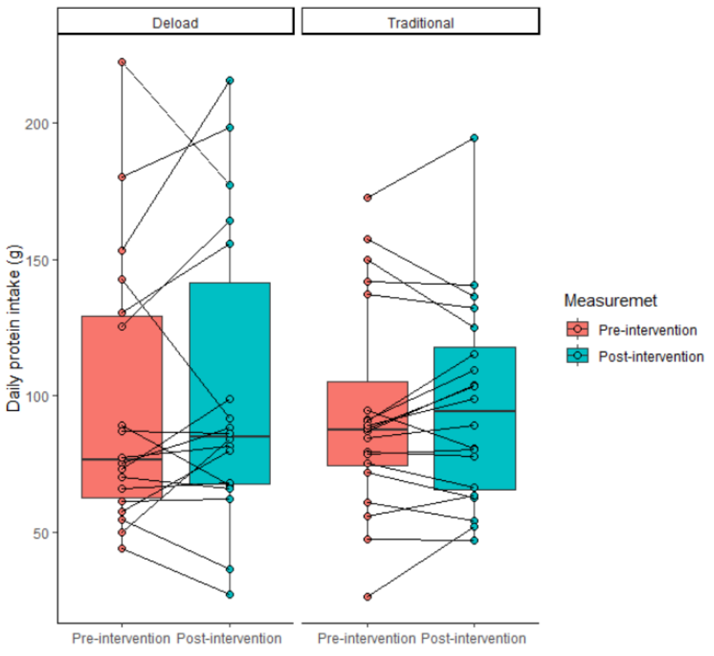

5) Assessment of potential differences

Variable	Multilevel: Time Difference [95%CrI]	Posterior probability increase post-intervention	Multilevel: Group Difference [95%CrI]	Posterior probability increase deload	ANCOVA: Group Difference [95%CrI]	Posterior probability increase deload
**Total energy intake (kcal)**	7.4 [-105 to 120]	p = 0.555	-26.1 [-475 to 470]	p = 0.458	-16.3 [-248 to 206]	p = 0.442
**Carbohydrate intake (gs)**	-2.8 [-19.8 to 13.1]	p = 0.357	6.1 [-57.4 to 72.6]	p = 0.579	2.5 [-30.6 to 35.8]	p = 0.559
**Fat intake (gs)**	0.41 [-5.2 to 6.1]	p = 0.570	-5.7 [-21.6 to 9.9]	p = 0.229	-4.3 [-14.9 to 6.5]	p = 0.216
**Protein intake (gs)**	3.7 [-3.8 to 11.2]	p = 0.851	4.3 [-23.1 to 33.2]	p = 0.623	2.4 [-12.6 to 16.9]	p = 0.621

Time difference represents the estimated difference between pre- and post-intervention across both groups. Group difference represents the estimated difference between the deload and traditional group.

Readiness to train

To assess participants' subjective feelings toward training across the study period a readiness-to-train questionnaire comprising 7 questions using Likert-type scales ranging from 1 to 4, 1 to 5 and 1 to 10 was given to participants 24-48 hours after the fourth and ninth weeks of the study. The upper and lower limit were anchored by the following sentence: "1 can be described as not at all/extremely low and 4, 5, 10 (depending on lower/upper end of the scale) can be described as extreme amount/extremely high."

Individual data points

1) Do you feel physically strong today? (Likert: 1 to 5)

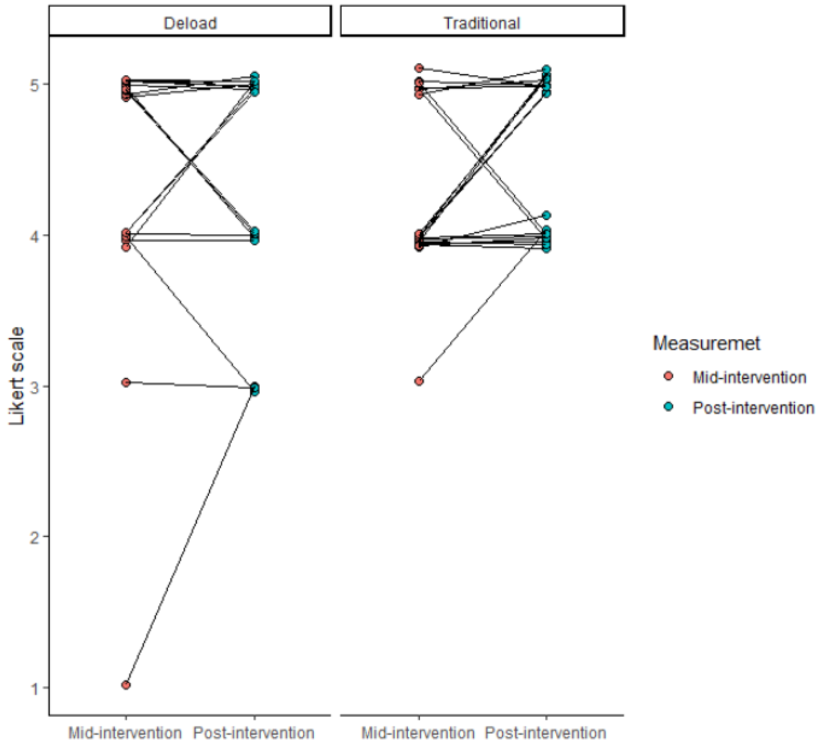

2) Do you feel mentally strong today? (Likert: 1 to 5)

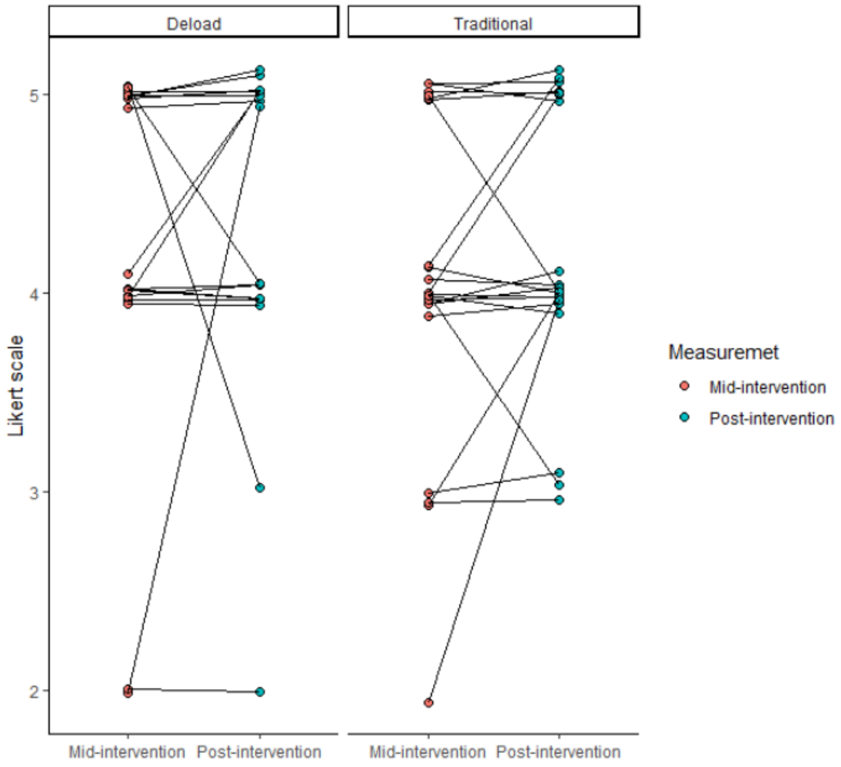

3) How would you describe your health today? (Likert: 1 to 4)

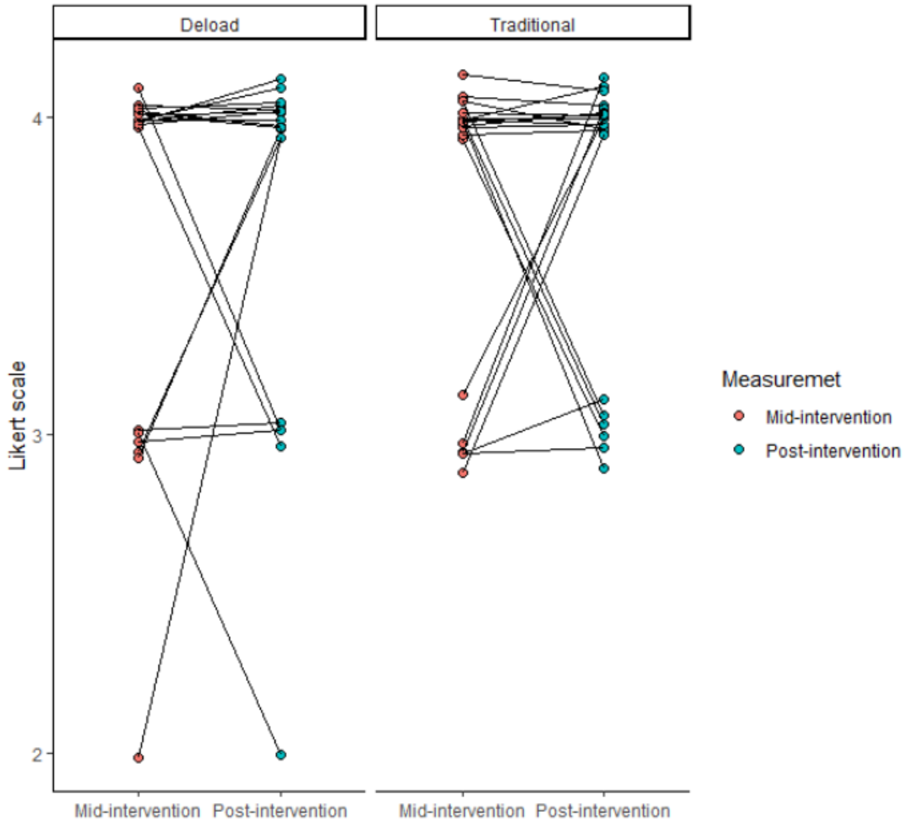

4) How would you describe your appetite today? (Likert: 1 to 5)

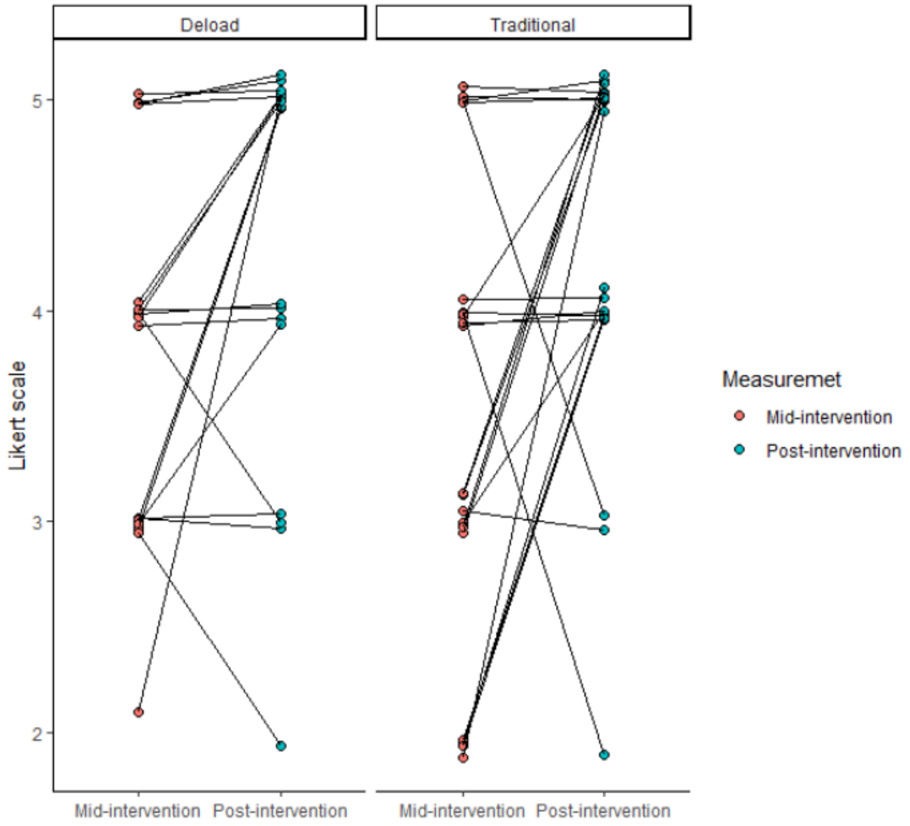

5) How would you describe your sleep quality over the last 24H? (Likert: 1 to 4)

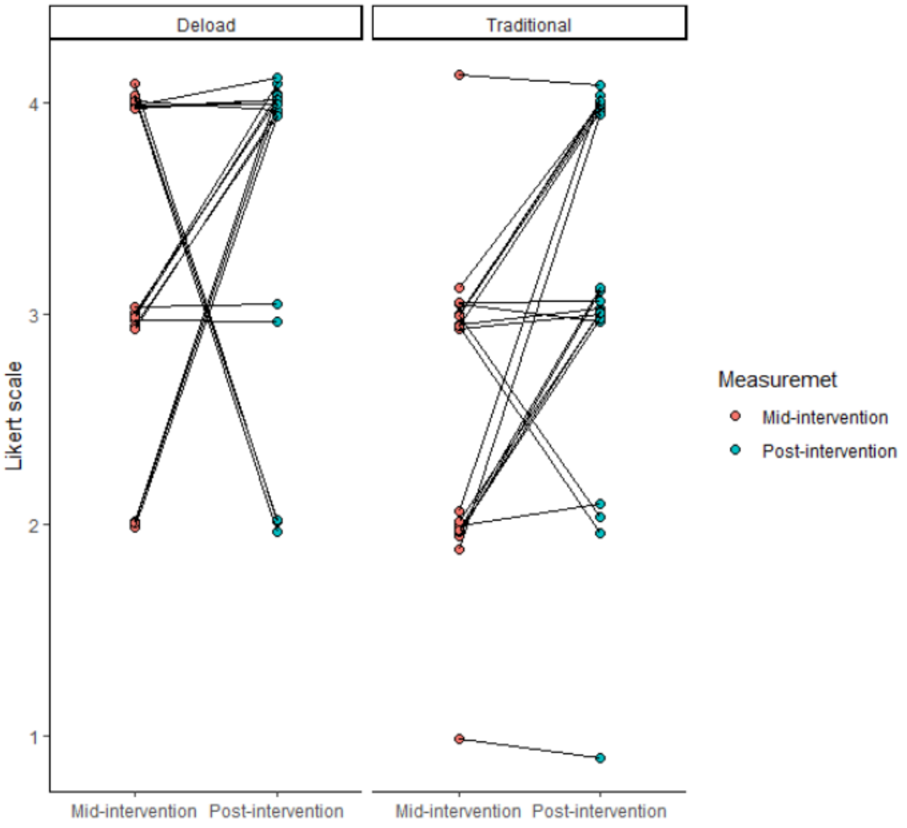

6) Do you have any muscle soreness today? (Likert: 1 to 10)

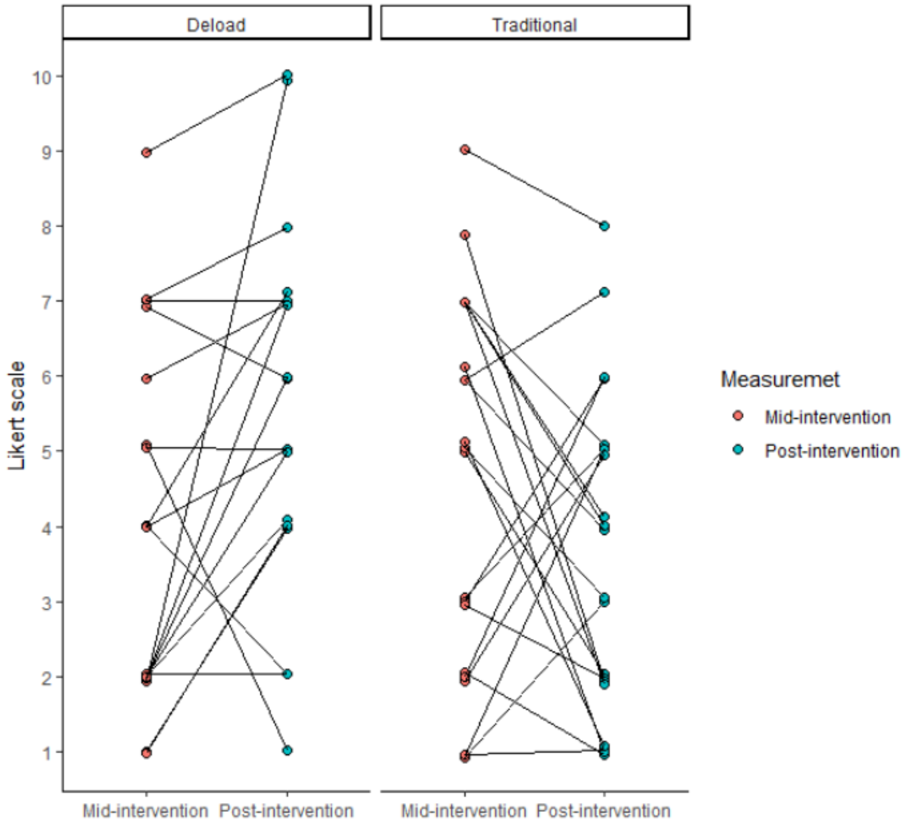

7) Rate your motivation to train today. (Likert: 1 to 10)

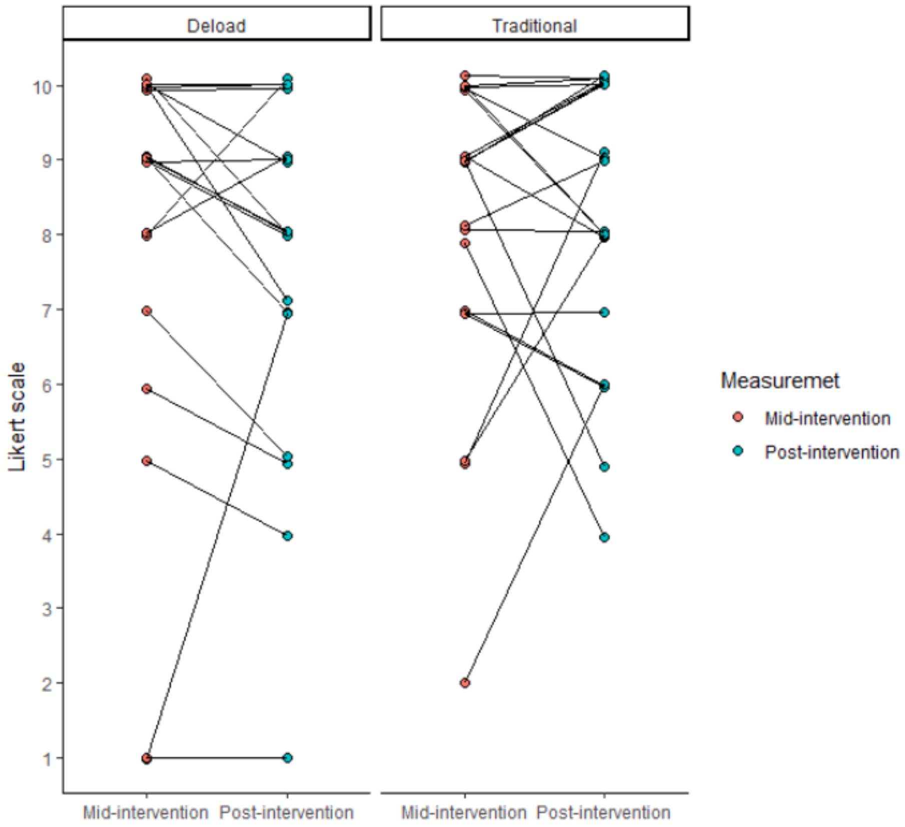

8) Assessment of potential differences

Variable	Mid-intervention: Group Difference [95%CrI]	Posterior probability increase deload	Post-intervention: Group Difference [95%CrI]	Posterior probability increase deload	ANCOVA: Group Difference [95%CrI]	Posterior probability increase deload
**Feel physically strong**	0.04 [-0.49 to 0.60]	p = 0.567	-0.13 [-0.58 to 0.33]	p = 0.261	-0.15 [-0.52 to 0.23]	p = 0.209
**Feel mentally strong**	0.18 [-0.39 to 0.77]	p = 0.727	0.15 [-0.36 to 0.64]	p = 0.616	0.07 [-0.38 to 0.52]	p = 0.623
**Describe your health**	-0.11 [-0.46 to 0.25]	p = 0.279	-0.05 [-0.40 to 0.29]	p = 0.387	-0.03 [-0.37 to 0.31]	p = 0.440
**Describe your appetite**	0.21 [-0.47 to 0.89]	p = 0.740	-0.01 [-0.60 to 0.58]	p = 0.488	-0.04 [-0.61 to 0.55]	p = 0.440
**Sleep quality last 24H**	0.70 [0.23 to 1.2]	p = 0.999	0.42 [-0.13 to 0.97]	p = 0.938	0.36 [-0.28 to 0.97]	p = 0.976
**Muscle soreness**	-0.58 [-2.2 to 1.0]	p = 0.244	1.9 [0.37 to 3.4]	p = 0.992	2.0 [0.53 to 3.5]	p = 0.995
**Motivation to train**	-0.30 [-1.9 to 1.3]	p = 0.459	-0.65 [-2.0 to 0.76]	p = 0.169	-0.50 [-1.6 to 0.66]	p = 0.193

Group difference represents the estimated difference between the deload and traditional group (deload:traditional).
